# Step-Wise Ethanol Adaptation Drives Cell-Wall Remodeling and *ROM2/KNR4* Activation in *Brettanomyces bruxellensis*

**DOI:** 10.3390/microorganisms13071489

**Published:** 2025-06-26

**Authors:** Leslie Hernandez-Cabello, Nachla Rojas-Torres, Liliana Godoy, Camila G-Poblete, Yarabi Concha, Verónica Plaza, Luis Castillo, Héctor M. Mora-Montes, María Angélica Ganga

**Affiliations:** 1 Departamento en Ciencia y Tecnología de los Alimentos, Universidad de Santiago de Chile, Alameda 3363, Santiago 9170022, Chile; 2 Departamento de Fruticultura y Enología, Facultad de Agronomía y Sistemas Naturales, Pontificia Universidad Católica de Chile, Santiago 8320165, Chile; 3 Laboratorio de Biología Molecular y Bioquímica, Departamento de Biología, Universidad de La Serena, La Serena 1700000, Chile; 4 Departamento de Biología, División de Ciencias Naturales y Exactas, Universidad de Guanajuato, Noria Alta s/n, Col. Noria Alta, C.P., Guanajuato 36050, Mexico

**Keywords:** *Brettanomyces bruxellensis*, cell wall integrity, spoilage wine yeast, ethanol adaptation

## Abstract

*Brettanomyces bruxellensis* has been described as the main spoilage microorganism in wines due to its ability to produce volatile phenols, which negatively impact the final product’s organoleptic properties. This yeast can grow and survive in environments that are too nutritionally poor and stressful for other microorganisms, and one of the stressful conditions it can endure is the high alcohol content in wine. In this study, cell wall morphology and the expression of some genes related to its composition were characterized under increasing ethanol concentrations to establish a possible ethanol resistance mechanism. *B. bruxellensis* LAMAP2480 showed greater resistance to β-1,3-glucanase activity when grown in media supplemented with 5% or 10% ethanol compared with the control assay (without ethanol). Transmission electron microscopy showed no significant differences in cell wall thickness during the different adaptation stages. However, the amount of wall polysaccharides and chitin briefly increased at 1% ethanol but returned to baseline at 5% and 10%. The amount of wall-associated protein increased progressively with each increment in ethanol concentration. In addition, overexpression of the *ROM2* and *KNR4/SMI1* genes was observed at 10% ethanol. These results suggest that the integrity of the cell wall might play an important role in the adaptation of *B. bruxellensis* to an ethanol-containing medium.

## 1. Introduction

The *Brettanomyces* genus of yeast was first isolated in the British brewing industry, being called Brettanomyces after “British fungus” [1]. Several species of this genus have been recognized, such as *Brettanomyces custersianus, Brettanomyces naardenensis, Brettanomyces nanus, Brettanomyces anomalus*, and *Brettanomyces bruxellensis*, the latter being the predominant one in wines [2,3]. *Brettanomyces bruxellensis* can degrade hydroxycinnamic acids, compounds naturally present in fruits with antimicrobial properties, into volatile substances, removing them from the grape must and thus not affecting its growth [2]. The degradation of these acids produces phenolic aromas, described as animal and horse sweat, among others, known as “Brett flavor” [4]. These sensory qualities cause deterioration of the product, which is reflected in a significant economic impact [5]. One of the most descriptive characteristics of *B. bruxellensis* is its ability to adapt to stressful conditions where other yeasts cannot proliferate [6]. For example, this species exhibits remarkable resistance to environments with high alcohol concentrations and limited availability of residual sugars and nitrogen sources. Its adaptation to elevated levels of sulfur dioxide (SO_2_), a chemical compound widely used in the food industry, is facilitated by mechanisms such as active sulfur reduction and efflux, enhanced acetaldehyde production, and the capacity to enter a viable but non-culturable (VBNC) state [7,8]. Industrially, *B. bruxellensis* is detected in the wine maturation process when the product rests in oak barrels, where the ethanol concentration is above 10% (*v*/*v*) [9]. For most microorganisms, a high alcohol concentration alters their growth due to an inhibition of cell division and decreases their cell volume and growth rate. Ethanol also influences cell metabolism and macromolecular biosynthesis by inducing the production of heat shock-like proteins, lowering the rate of RNA and protein accumulation, enhancing the frequency of petite mutations, altering metabolism, denaturing intracellular proteins and glycolytic enzymes, and reducing their activity [10]. The cell wall is the first physical barrier of microorganisms that is affected by any external environmental change [11]. This structure protects against mechanical stress since the combination of the strength and elasticity of the cell wall provides an effective barrier against compression. Likewise, it protects against osmotic shock when there is rapid exposure.

On the other hand, the cell wall is required to establish and maintain the cell shape, which is essential for forming a new cell and its division [12]. The yeast cell wall constitutes 15–30% of its cellular dry weight and 25–50% of its volume, comprising β-1,3 glucan (240 kDa), β-1,6 glucan (24 kDa), mannan bound with protein (100–200 kDa), and chitin (25 kDa) [12]. The composition of the cell wall depends on the strain, culture conditions, physiological state, and growth stage of the yeast [12]. 

In *Saccharomyces cerevisiae,* two mechanisms have been described that play a central role in cell wall biogenesis, maintenance, and stress resistance: the cell wall integrity (CWI) pathway and the calcineurin (CN) pathway [13]. CWI is the most studied, and it is activated by a set of plasma membrane-spanning sensors through activation of the cell surface sensor proteins Wsc1-3 and Mtl1 in the face of a stress factor [14]. Surface sensors act as linear nano springs that transmit or detect damage or stress in the cell wall to continuous receptors in the signaling pathway [15]. Cell surface sensors activate Rom2p, a guanine nucleotide exchange factor for the GTP protein (GEP) encoded by the *ROM2* gene [16]. The GTP protein is responsible for activating the G-protein and performing the transduction of intracellular signals, such as growth regulation and stress response, in addition to contributing to the activation of the Pkc1 protein [14,16]. This protein plays a fundamental role in the CWI pathway since, among other functions, it allows the activation of the mitogen-activated protein (MAP) kinase cascade of cellular integrity PKC (MAPK) and the subsequent factors that carry out transcription (Rlm1). In this way, it activates the function of the *SED1* gene, which encodes the structural glycoprotein GPI [16].

Alternatively, Smi1, the protein encoded by the *KNR4/SMI1* gene, appears to relay stress signals by engaging the MAPK cascade, which in turn activates the Rlm1p transcription factor; this protein is also associated with regulating the glucan content of the cell wall [15,17].

In the case of *B. bruxellensis,* some mechanisms that are involved in its resistance to ethanol have been described. Brettanomyces species, including *B. bruxellensis,* are Crabtree-positive, capable of fermenting glucose to ethanol even in the presence of oxygen when glucose is abundant [18]. This fermentative capacity may contribute to their persistence in alcoholic environments. Montagner et al. [19] reported that ethanol concentrations could affect cells’ surface properties, affecting adhesion proteins, thus facilitating their bioadhesion capacity. Recently, Di Canito et al. [20] demonstrated that their flocculent character and greater adhesiveness could allow for better survival of *B. bruxellensis* under stress conditions. It is interesting to know how species of the *Brettanomyces* genus can adapt to high ethanol concentrations during alcoholic fermentation.

Here, we exposed *B. bruxellensis* LAMAP2480 to a defined range of ethanol concentrations to (i) measure cell wall modifications with zymolyase treatment and microscopy, and (ii) track expression changes in key cell wall integrity pathway genes. The results showed that the yeast adapts to wine-like ethanol levels by reorganizing its cell wall structure.

## 2. Materials and Methods

### 2.1. Media and Growth Conditions

The *Brettanomyces bruxellensis* strain LAMAP 2480, held in the Biotechnology and Applied Microbiology Laboratory at the Universidad de Santiago de Chile, was maintained on YPD agar (20 g/L glucose, 5 g/L yeast extract, 5 g/L peptone, 20 g/L agar) and incubated at 28 °C for 96 h to obtain fresh colonies. A single colony was transferred to 5 mL of YPD broth and cultivated for 72 h at 28 °C with orbital agitation (250 rpm). This starter culture served as the inoculum for 100 mL of fresh YPD broth adjusted to an initial density of 1 × 10^6^ cells/mL (optical density at 600 nm (OD_600_) 0.1) and incubated for a further 48 h under identical conditions, thereby establishing the ethanol-free reference culture (0% *v/v* ethanol).

Progressive adaptation to ethanol was achieved through three successive transfers, each conducted at 28 °C and 250 rpm. First, cells from the reference culture were inoculated at 1 × 10^6^ cells m/L into a 100 mL medium composed of 90 mL YPD and 10 mL synthetic wine (SW), yielding a final ethanol concentration of 1% (*v*/*v*). The SW formulation (0.6 g/L glucose, 1.2 g/L fructose, 0.3 g/L trehalose, 2 g/L yeast extract, 1 g/L (NH_4_)_2_SO_4_, 0.4 g/L MgSO_4_·7H_2_O, and 2 g/L KH_2_PO_4_ in 10% *v/v* ethanol) was obtained from Coronado et al. [21]. After 8 h, 1 × 10^6^ cells/mL were harvested and transferred into 100 mL medium composed of 50 mL YPD mixed with 50 mL SW, raising the ethanol level to 5% (v/v), and incubated for another 8 h. Finally, 1 × 10^6^ cells/mL from the previous medium were harvested and inoculated into 100 mL of SW + 10% (*v*/*v*) ethanol condition and incubated for 24 h.

Cell concentrations were verified at each transfer by Neubauer chamber (Precicolor, HBG, Giessen-Luetzellinden, Germany) counts. This step wise regimen produced a population incrementally acclimated to 10% ethanol while minimizing physiological shock and allowing reproducible comparisons across all adaptation stages.

### 2.2. Cell Wall Susceptibility Analysis

Structural alterations in the cell wall of *B. bruxellensis* LAMAP 2480 that arose during step wise ethanol adaptation were evaluated by determining in each culture the cell wall susceptibility to the lytic enzyme zymolyase 20T (AMS Biotechnology, Milton, UK), following Shimoi et al. [22]. For every adaptation stage, 50 mL of culture was harvested under centrifugation at 6000 rpm for 30 min. The resulting pellet was rinsed once with sterile distilled water and resuspended in 1.3 mL of 0.1 M sodium phosphate buffer (pH 7.5). Zymolyase was then added to give a final concentration of 20 µg/mL. Cell lysis was monitored spectrophotometrically as the time-dependent decrease in optical density at 600 nm (OD_600_); the reduction in OD_600_ relative to the initial reading was taken as a quantitative measure of cell wall susceptibility.

### 2.3. Transmission Electron Microscopy (TEM)

To observe changes in the cell wall during the different stages of adaptation to ethanol, 5 mL of culture was centrifuged at 6000× *g* for 5 min. Then, the pellet obtained was washed with sterile distilled water twice. Subsequently, the pellet was fixed with 2.5% glutaraldehyde in 0.1 M sodium cacodylate buffer pH 7.0. As a control, yeast were grown in YPD medium. Pontificia Universidad Católica de Chile provided the TEM service.

### 2.4. Polysaccharide Composition of the Cell Wall

The polysaccharides were quantified by Dionex liquid chromatography, using glucose, mannose, and glucosamine as standard patterns. The service was provided by Dr. Luis Castillo from the Universidad de La Serena jointly with the Universidad de Guanajuato, Mexico. Cells were collected by centrifugation at 8500× *g* for 10 min, washed twice with 50 mM sodium phosphate buffer (pH 6.0), and broken with glass beads using a FastPrep machine (Qbiogene, Irvine, CA, USA). The homogenate was centrifuged at 21,500× *g* for 10 min. The pellet was washed with 1 M NaCl, resuspended in buffer (500 mM Tris-HCl buffer [pH 7.5], 2% (wt/vol) SDS, 0.3 M β-mercaptoethanol, 1 mM EDTA), boiled for 10 min, and freeze-dried [23]. For glucose and mannose quantification, cell walls were hydrolyzed in 2 M trifluoroacetic acid, boiled for 3 h, washed, and centrifuged at 21,500× *g* for 10 min. Chitin content was determined by hydrolyzing the cell walls in 6 N HCl at 100 °C for 17 h. Quantification of the sugar monomers from the acid-hydrolyzed walls was achieved by high-performance anion exchange chromatography with pulsed amperometric detection in a carbohydrate analyzer from the Dionex-LC system (Surrey, UK) [23]. The total protein concentration was determined using the Bradford method [23,24].

### 2.5. Evaluation of Some Genes Overexpressed in B. bruxellensis Under Different Ethanol Concentrations

Previously, our group identified the overexpression of orthologous genes of *S. cerevisiae* in *B. bruxellensis* when this latter yeast was grown in 10% ethanol. These genes included *ROM2*, *SED1,* and *KNR4/SMI1*. Then, in this research, we continued to deepen our study. The nucleotide sequences of the *KNR4/SMI1*, *ROM2*, and *SED1* genes of *S. cerevisiae* S288c, as well as the amino acid sequences of the proteins encoded by the genes of interest, were obtained from the KEGG database (https://www.genome.jp/kegg/, accessed on 17 May 2024) of the Bioinformatics Center, Institute for Chemical Research, Kyoto University. Genomic data of the *B. bruxellensis* strain LAMAP2480 were obtained from the National Center for Biotechnology Information (NCBI) database (http://www.ncbi.nlm.nih.gov, accessed on 17 May 2024). Local tBLASTn alignments were performed between the amino acid and nucleotide sequences of *S. cerevisiae* and *B. bruxellensis* LAMAP2480 to determine a sequence of interest and domains related to proteins involved in the cell wall integrity pathway.

From the alignments performed, primers were designed for the *KNR4/SMI1, ROM2,* and *SED1* genes using the NCBI database (Table 1).

### 2.6. RNA Extraction

RNA was extracted from the samples described in point 2.1, according to [25] Godoy et al. (2016). At the end of the *lag* phase, 50 mL aliquots were taken and then centrifuged at 2850× *g* for 10 min. The pellet was resuspended in 200 μL of RNA buffer (50 mM Tris-HCl pH 7.4, 100 mM NaCl, 10 mM EDTA), 400 μL of acidic phenol (Winkler, Chile) and glass beads previously washed with HCl. Vortex agitation was then repeated 3 times for 1 min each, and incubation on ice for 1 min. Then, 200 μL of RNA buffer and 40 μL of 10% SDS were added. The samples were incubated for 6 min at 65 °C and centrifuged at 16,000× *g* for 15 min at 4 °C. The precipitate was then discarded, transferring the supernatant to a new tube, to which 400 μL of acid phenol and 40 μL of 3 M sodium acetate were added. It was then centrifuged at 16,000× *g* for 15 min at 4 °C. The supernatant was transferred to a new Eppendorf tube, and then 1 mL of cold 96% ethanol was added and left for 2h at −80 °C. The precipitated RNA was centrifuged at 4 °C for 10 min and then continued with the PureLink RNA Mini Kit from Invitrogen Thermo Fisher Scientific (Waltham, MA, USA). The integrity of the RNA was assessed by 1% agarose gel electrophoresis.

### 2.7. Relative Expression Quantification

For RT-PCR, the RQ1 RNase-Free DNase (Promega, Madison, WI, USA) and M-MLV RT (Promega, USA) protocols were used. The qPCR assays were performed on AriaMx Real-Time PCR equipment (Agilent Technologies, Petaling Jaya, Malaysia) using Agilent Aria 1.8 equipment software. The reactions were performed in a final volume of 20 µL according to the 5x HOT FIREpol EvaGreen qPCR Mix Plus (ROX) protocol (Solis Biodyne, Tartu, Estonia) with the following program: 95 °C for 12 min, 40 amplification cycles of 95 °C for 15 s, 50.5 °C to 55.2 °C (according to Tm primers, Table 1) for 20 s and 72 °C for 20 s. In addition, the dissociation curve at the end of the qPCR cycle was performed with the following program: 95 °C for 15 s, 50.5 °C to 55.2 °C for 1 min, and 95 °C for 15 s.

Each reaction was performed in triplicate for each gene under study, using *ACT1* as a reference gene (housekeeping) [26]. Relative quantification of the genes of interest was performed using the mathematical method described by Livak and Schmittgen [27]. This approach evaluates relative changes in gene expression between an experimental condition (synthetic wine with ethanol) and a control condition (YPD medium, ethanol-free reference culture) normalized to an internal reference or housekeeping gene.

To determine the efficiency (E) of the reaction and the correlation coefficient (R^2^) for the amplifications performed by each gene under study, the linear regression model estimated by Svec et al. [28] was used.

### 2.8. Statistical Analysis

Relative gene expression levels were quantified using the *t*-test. One-way ANOVA (*p* < 0.05) followed by Duncan’s multiple range test was used to analyze the effects of zymolyase on the cell wall of *B. bruxellensis*, as well as for the transmission electron microscopy (TEM) and the quantification of cell wall polysaccharide content. Statistical analyses were performed using Statgraphics Plus v.19 software (Manugistic Group, Inc., Rockville, MD, USA). Graphs were constructed using GraphPad Prism v.10 software (Boston, MA, USA). All experiments were conducted in triplicate.

## 3. Results

### 3.1. Effect of the Zymolyase Enzyme on the Cell Wall of B. bruxellensis Grown at Increasing Ethanol Concentrations

**Figure 1:**
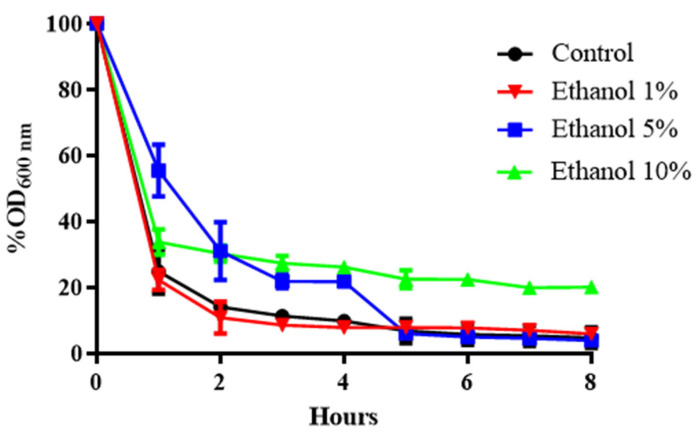
Susceptibility of the cell wall of *B. bruxellensis* to the enzyme zymolyase in a sample of yeast grown at different ethanol concentrations. Comparison among cells grown with 1%, 5%, and 10% ethanol. Control: YPD medium. The experiments were performed in triplicate. Statistical analysis was performed using one-way ANOVA (*p* < 0.05), followed by Duncan’s multiple range test.

The commercial enzymatic preparation known as zymolyase contains several enzymatic activities that can attack different cell wall polymers. The main one is β-1,3 glucan laminaripentaohydrolase, which hydrolyses linear glucose polymers with β-1,3 linkages and residual protease activity [29,30]. To determine whether the concentration of β-glucans in the yeast cell wall is affected by growth in ethanol medium, *B. bruxellensis* cultures grown at different concentrations of this alcohol were exposed to a 20 μg/mL solution of zymolyase 20T (Figure 1).

Figure 1 shows that as the ethanol concentration in the culture medium of *B. bruxellensis* increases, the sensitivity to zymolyase decreases. After 2 h at 1% (*v*/*v*) ethanol, survival dropped by approximately 90%. When ethanol increased to 5% or 10% (*v*/*v*), survival fell by approximately 70% (Figure 1). Likewise, when the cell was previously adapted to ethanol, it was observed that there was greater resistance to exposure to zymolyase (Figure 1). This suggests that when yeast is grown in the presence of alcohol, a change in the composition of glucose polymers with β-1,3 linkages could occur, the links that zymolyase degrades.

### 3.2. Analysis of the Cell Wall by TEM

**Figure 2:**
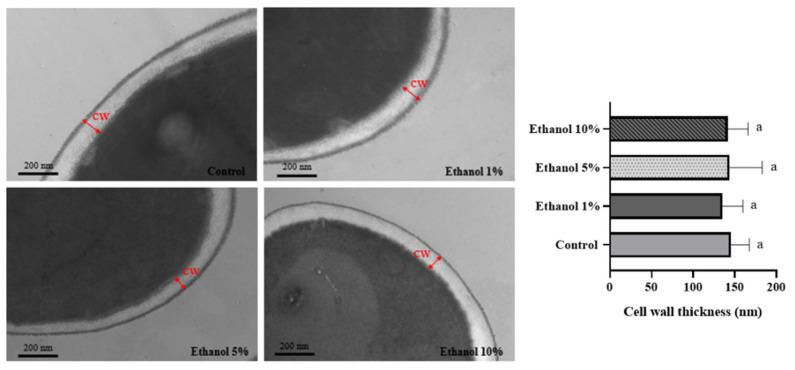
TEM of *B. bruxellensis* during the endpoint of the *lag* phase of the growth curve. Representative images of untreated cells and cells treated with different concentrations of ethanol are shown. Cell wall thickness analysis was measured by taking 11 different points from six cells in each treatment and control. Treatments: synthetic wine (SW) with ethanol 1%, 5%, and 10%. Control: yeast grown in YPD medium. CW: cell wall. The experiments were performed in triplicate. Statistical analysis was performed using one-way ANOVA (*p* < 0.05), followed by Duncan’s multiple range test. Different letters in the same graphic indicate statistically significant differences (*p* < 0.05).

Transmission electron microscopy shows that yeast, such as *S. cerevisiae* and *Kluyveromyces lactis*, have a cell wall formed by two layers. The chitin, β-1,3, and β-1,6 glucans composition is responsible for the electron-transparent inner layer and gives it its rigidity. The dense outer layer comprises mannoproteins. Most non-*Saccharomyces* yeasts can survive under extreme conditions such as high pH, high temperature, etc. [31], where the cell wall plays a crucial role in the mechanical properties. TEM analysis allowed us to determine the cell wall thickness of *B. bruxellensis* yeasts grown in different ethanol concentrations. Figure 2 shows the results obtained, observing that the cell wall thickness was similar in all the samples analyzed.

### 3.3. Determination of Cell Wall Polysaccharide Concentration

**Figure 3:**
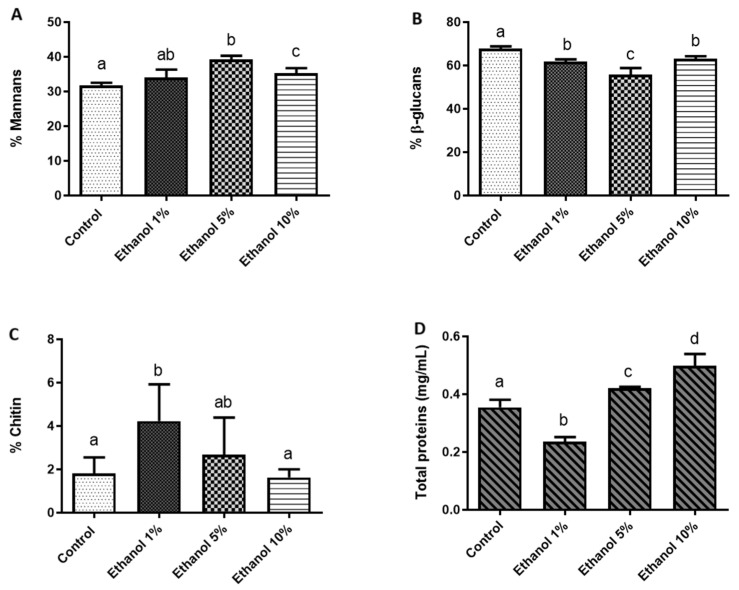
Percentage of polysaccharides and cell wall proteins of *B. bruxellensis.* (**A**) Mannans, (**B**) glucans, (**C**) chitin, and (**D**) total proteins (mg/mL). All quantifications were performed in triplicate. Statistical analysis was performed using one-way ANOVA (*p* < 0.05), followed by Duncan’s multiple range test. Different letters in the same graphic indicate statistically significant differences (*p* < 0.05).

To quantify the components that constitute the cell wall of *B. bruxellensis* and determine whether yeast growth at different ethanol concentrations alters this chemical composition, a quantification of mannans, glucans, and chitin, as well as the protein content in the cell wall, was carried out (Figure 3).

Figure 3 shows the ethanol-dependent remodeling of the *B. bruxellensis* LAMAP2480 cell wall. In YPD medium (nutritive and ethanol-free medium), the wall was dominated by β-glucans (70% of the material quantified), while mannans accounted for 32%, chitin for 2%, and wall-associated proteins for 0.35%. The introduction of 1% (*v*/*v*) ethanol elicited an immediate redistribution of these components: β-glucans and total proteins declined, whereas the mannan fraction rose modestly and chitin nearly doubled, indicating an early reinforcement of the wall’s structural scaffold. At 5% ethanol, the trend shifted. Mannans and proteins surpassed the control values, β-glucans underwent a further reduction, and chitin returned to levels indistinguishable from the YPD reference. This pattern suggests compensatory synthesis of mannoproteins to counterbalance the progressive loss of β-glucan polymers. Growth in 10% ethanol accentuated the same response. β-glucans remained significantly lower than in control (YPD), yet both mannans and proteins exceeded control levels, reflecting a continued bias toward a more mannoprotein-rich, β-glucan-poor wall architecture. Chitin again showed no detectable difference from the control, implying that its earlier increase was transitory and confined to the initial ethanol shock.

### 3.4. Obtaining the orthologous genes KNR4/SMI1, ROM2 and SED1 from S. cerevisiae in B. bruxellensis

Yeast cells sense and respond to physiological stress by evoking an adaptive response. Ethanol is an important inhibitor of yeast growth that works at relatively low concentrations. It inhibits cell division and decreases cell volume and specific growth rates, while a high ethanol concentration reduces cell vitality and increases cell death [32]. Ethanol also influences cell metabolism and macromolecular biosynthesis by inducing the production of heat shock-like proteins, lowering the rate of RNA and protein accumulation, enhancing the frequency of petite mutations, altering metabolism, denaturing intracellular proteins and glycolytic enzymes, and reducing their activity [10]. There is a well-studied signal transduction cascade called cell wall integrity (CWI), which is activated by a set of plasma membrane-spanning sensors in yeast. Some genes activated with increased ethanol concentration in the culture medium have been described.

An example is two genes that code for the Rom1/Rom2 factors (guanine nucleotide exchange factors), which activate the small-G protein Rho1 [33]. It has been demonstrated that *ROM2* is required for *S. cerevisiae* growth in an ethanol-containing medium and is involved in cell wall biosynthesis [34]. In addition, our research group carried out an experiment where *B. bruxellensis* was grown in SW with 10% ethanol. Under these conditions, we identified an overexpression of orthologous genes of *S. cerevisiae* in *B. bruxellensis*. Some of these genes were *ROM2*, *SED1*, and *KNR4/SMI1*. Likewise, a change in the expression of the *SED1* gene, which is involved in the metabolism of a glycoprotein, has been described, as well as *KNR4/SMI1*, which is related to the increase in β-glucan in the cell wall and its assembly [11].

The first genomes of *B. bruxellensis* were described in the 2000s [35]. Although some genes are already annotated in this genome, they are not yet fully described. Therefore, to evaluate the effect of ethanol on the expression of the genes of interest, it was necessary to suggest a possible location and sequence of *KNR4/SMI1*, *ROM2*, and *SED1*, orthologues of *S. cerevisiae* S288c in *B. bruxellensis* LAMAP2480. For this purpose, the nucleotide and amino acid sequences of these genes in *S. cerevisiae* S288c were searched using the KEGG database (https://www.genome.jp/kegg, accessed on 17 May 2024). To obtain suggestions for the possible location of the genes of interest in the *B. bruxellensis* LAMAP 2480 genome, a tBLASTn alignment was performed at NCBI with the amino acid sequences of the proteins encoded by the *KNR4/SMI1, ROM2,* and *SED1* genes. In this way, the areas with the highest percentage of identity in the analyzed sequence were identified (Table 2).

In the case of the *KNR4/SMI1* gene, the database identified four possible similar regions in the genome sequence of *B. bruxellensis* LAMAP2480, with a level of gene coverage in the genome of 67%, with an identity percentage of 30.62%. This alignment showed the highest level of identity, which was optimal for the research objective. As for the *ROM2* gene, two segments were obtained with a high level of homology between the amino acid sequences of the gene and the *Brettanomyces* genome. The identity percentage between the gene and the genome segment was 45.08%, with a total coverage of 69%. For the *SED1* gene, the encoded amino acid sequences of this gene were analyzed, obtaining a similarity between two genome segments. By identifying the possible sequences of the *KNR4/SMI1, ROM2*, and *SED1* genes in *B. bruxellensis,* primers were developed for subsequent experiments.

### 3.5. Gene Expression Analysis

**Figure 4:**
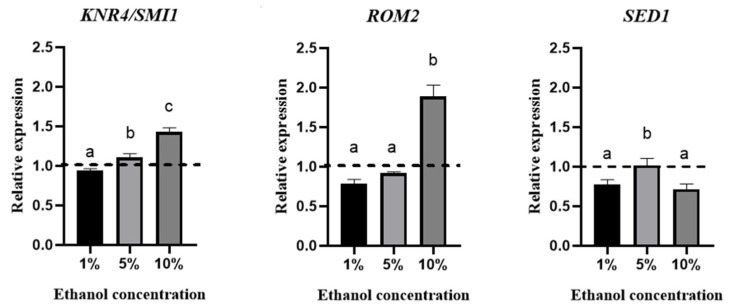
Relative expression of *KNR4/SMI1*, *ROM2,* and *SED1* genes at the endpoint of the *lag* phase at different ethanol concentrations (1%, 5%, and 10%). The line above 1 is considered overexpression. The experiments were performed in triplicate, and statistical analysis was performed using t tests. Different letters in the same graphic indicate statistically significant differences (*p* < 0.05).

The effect of ethanol concentration on the relative expression of these genes was evaluated using qPCR (Figure 4).

This figure shows overexpression of the KNR4/SMI1 gene in culture media containing 5% and 10% (*v*/*v*) ethanol. In the case of the *ROM2* gene, overexpression was only observed when the yeasts were grown in the presence of 10% (*v*/*v*) ethanol. No overexpression was observed for the *SED1* gene under the conditions studied.

## 4. Discussion

The wine fermentation process begins with a varied population of yeasts selected as the alcohol concentration increases. This is how a few yeast genera remain active in substrates with concentrations above 10% (*v*/*v*) of ethanol. Adapting these genera to high ethanol concentrations is crucial for their survival. Several studies have reported that the cell wall is essential since changes in cell composition and loss of rigidity have been observed when subjected to high alcohol concentrations [11,36]. The cell wall is the first physical barrier against the different culture conditions for yeasts and is essential for cell survival [37].

For *B. bruxellensis*, there are no studies that have analyzed the composition of its cell wall or the possible adaptation mechanisms of this yeast to high alcohol concentrations, considering that this microorganism usually proliferates during the maturation of wines, where there is at least 10% (*v*/*v*) alcohol [5].

In the present work, the structural changes in the cell wall and CWI-related gene expression in cells grown in the presence of ethanol were evaluated. First, a sensitivity test was performed with the enzyme zymolyase, which has β-1,3 glucanase activity (Figure 1). The results indicated a lower sensitivity of the cell wall to the activity of this enzyme as the ethanol concentration increased. Similar results have been described in *S. cerevisiae* when it was grown in a medium supplemented with 6% (*v*/*v*) ethanol, observing a lower sensitivity to β-1,3 glucanase activity [38]. This suggests that the cell wall of both *S. cerevisiae* and *B. bruxellensis* changes its chemical composition as the ethanol concentration in the culture medium increases, resulting in an adaptation mechanism to this stress factor.

On the other hand, it has been described that in *S. cerevisiae,* no significant changes in cell wall thickness are observed in cells grown in a medium supplemented with 9% (*v*/*v*) ethanol [36]. A similar observation was made in the present study (Figure 2). This would indicate that there are no changes in the spatial configuration of the cell wall components when varying the culture medium that the yeast faces. The yeast cell wall is organized into two main layers, composed of four macromolecules: cell wall proteins, β-1,6 glucans, β-1,3 glucans, and chitin [39]

To evaluate whether ethanol stress reshapes the cell wall architecture, we quantified the main polysaccharide fractions. Rising ethanol concentrations led to a drop in chitin and a concomitant rise in total protein content (Figure 3). Mannan levels also trended upward during adaptation, reaching a peak at 5% (*v*/*v*) ethanol. In the case of glucans, β-glucans stayed below control values even at 10% ethanol (Figure 3). Opposite results have been reported in *S. cerevisiae*, where there are no significant differences in the percentage of mannans, glucans, or chitin when grown in a medium supplemented with 9% (*v*/*v*) ethanol [40]. However, Orlean [41] indicated that an increase of ethanol in the culture medium leads to remodeling of the cell wall architecture to allow it to become more robust. This includes an increase in cell wall components and a change in the cross-linking between them. Some mannoproteins can have a structural role or mediate social activity by serving as mating agglutinins, or they might promote the formation of biofilms. Mannoprotein would have a role in yeast adaptation to this stress [42]. On the other hand, Uscanga and Francois [39] demonstrated that cell growth with ethanol was almost completely refractory to zymolyase, indicating it might produce more β-1,6 glucan than β-1,3 glucan. β-1,6 glucan will be an important component of the cell wall in stress conditions. Bekirian et al. [43] showed that β-1,6 glucan has a pivotal role in determining the architectural arrangement of polysaccharides in the cell wall. This will influence growth, drug sensitivity, cell morphology, and filamentation. β-1,6 glucan biosynthesis is stimulated via a compensatory pathway when there is a defect in cell wall mannan biosynthesis. Then, the variation in the concentration of these two cell wall compounds in the *B. bruxellensis* cell wall is related to the mechanism that yeast use in stress conditions.

Our results suggest that *B. bruxellensis* would respond to ethanol differently from that observed in *S. cerevisiae.* Valdivia and Schekman [44] reported that *S. cerevisiae*, in response to heat, decreases the synthesis of β-glucans and increases the export of chitin synthases from chitosomes to the plasma membrane. Notably, the changes induced by ethanol are identical to those caused by heat stress, suggesting a “functional overlap” between heat- and ethanol-induced cell damage [45].

In previous work, we observed that when *B. bruxellensis* was grown in a medium containing ethanol, cell wall-related genes such as *ROM2, KNR4/SMI1,* and *SED1* were overexpressed. In the current study, we confirmed the overexpression of the *ROM2* gene (Figure 4) when the yeast was grown in a medium supplemented with 10% (*v*/*v*) ethanol. In this regard, genes required for growth in *S. cerevisiae* under ethanol stress have been identified, demonstrating that *ROM2* is essential for its growth [35]. It has been described that the Rom2p protein is fundamental for activating the CWI pathway and for the synthesis of β-glucans. *S. cerevisiae* mutants in the *ROM2* gene have reduced its β-1,3 glucan synthase activity [46]. Likewise, the overexpression of ROM2 in *S. cerevisiae* triggers the accumulation of β-1,3 glucan in secretory vesicles to be exported to the cell wall. Additionally, there is an increase in *ROM2* gene expression in yeast cells grown in a medium supplemented with 1 M sorbitol, defined as a polyalcohol [47]. Our results showed that when yeast grows with 10% (*v*/*v*) ethanol, there is a slight increase in β-glucans. However, this somewhat contradicts the sensitivity of *B. bruxellensis* cells to the enzyme zymolyase, which decreases when this microorganism grows in a medium supplemented with 10% (*v*/*v*) ethanol. Comparing different responses to different stressors reveals not only the existence of specific transcriptional adaptation profiles for each situation but also the presence of a standard signature that is induced in all these stress situations [29].

On the other hand, in *S. cerevisiae*, the Knr4/Smi1 protein represents a conserved family of fungus-specific proteins. It was initially identified in *Hansenula mrakii* during a study about genes affecting cell wall β-1,3 glucan synthesis. This gene would be related to increased chitin concentration and decreased β-glucans in the yeast cell wall [13]. The *KNR4/SMI1* gene has been described to encode a phosphoprotein in several cellular processes, such as cell wall maintenance, cell cycle, osmoregulation, and spore formation [48]. This gene is required to appropriately target the RIm1p and Swi4p transcription factors by the mitogen-activated protein kinase (MAPK) Slt2, which is involved in cell wall remodeling [13]. The overexpression of *KNR4/SMI1* in a wild-type *S. cerevisiae* strain caused increased resistance to drugs that affect the cell wall. Figure 4 shows the expression of the *KNR4/SMI1* gene when *B. bruxellensis* was grown in different concentrations of ethanol, observing that at both 5% and 10% ethanol there was an overexpression of this gene. A similar response was reported by *S. cerevisiae* when it was grown in a medium with 5% (*v*/*v*) of ethanol for 30 min, observing an overexpression of this gene [49].

In the case of the *SED1* gene, it has been described as necessary in cell wall integrity since it encodes the Sed1p protein, which is involved in the recovery of proteins from the endoplasmic reticulum of the secretory and structural pathway of the cell wall. This is the main stress-induced glycoprotein (GPI) [50]. In *S. cerevisiae*, the expression of *SED1* occurs mainly in the stationary phase of cell growth [22]. When the *SED1* gene was interrupted, it was observed that it does not affect the cell in the exponential growth phase, but in the stationary phase it is crucial for lytic resistance. By mutating this gene in *S. cerevisiae*, an increase in sensitivity to zymolyase was observed compared to the wild-type strain [22].

On the other hand, the overexpression of *SED1* in *S. cerevisiae* cells exposed to 5% (*v*/*v*) ethanol for one hour was observed as a phenomenon of adaptation to ethanol during the first phase of the growth curve (*lag* phase) [51]. *SED1* is upregulated under thermal, oxidative, nutritional, and hyperosmotic stress. These findings suggest that *SED1* has an important function of protection against any factor that induces extreme conditions in the cell [12,22]. In the case of *B. bruxellensis*, overexpression of this gene was not observed when the yeasts were exposed to 1, 5, or 10% (*v*/*v*) ethanol. Moreover, there was repression in this last condition (10% ethanol). The expression of the *SED1* gene was not detected after the adaptation time of the growth curve of *S. cerevisiae* [51]. Our study considered the expression of these genes at the end of the *lag* phase, so it is important to consider the growth stage in a future study.

## 5. Conclusions

Our findings demonstrate that *B. bruxellensis* adapts to wine-like ethanol levels by strategically remodeling its cell wall rather than thickening it. A temporary increase in chitin was observed at 1% ethanol during a stepwise ethanol concentration increase from 0% to 10% (*v*/*v*), as shown by quantitative chromatography, followed by a return to its initial levels and concurrent rises by mannans of 20% and by wall-associated proteins of 60%. This compositional shift translated into functionally stronger walls: susceptibility to the β-1,3-glucanase zymolyase dropped by more than half, yet transmission electron microscopy confirmed that the wall thickness remained unchanged.

At the molecular level, we identified the *B. bruxellensis* orthologues of *ROM2* and *KNR4/SMI1* and observed ≥2-fold over-expression of both genes at 10% ethanol, while *SED1* expression stayed constant. This is the first evidence that the conserved cell wall integrity pathway operates in this species under ethanol stress. Together, the biochemical, structural, and transcriptomic data converge on a single adaptive mechanism: ethanol triggers a controlled depletion of chitin and enrichment of mannoproteins, orchestrated by the *ROM2/KNR4* axis, to reinforce the wall’s mechanical resilience and likely restrict ethanol ingress—ultimately enabling *B. bruxellensis* to thrive in high-ethanol environments.

## Figures and Tables

**Table 1:** Primers designed for qPCR analysis of the genes of interest.

Gene	Sequence (5′–3′)	Tm (°C)	Amplicon Length (bp)
*KNR4/SMI1*	F: CCTTGCAGGAGCAGAAATAC	55.0	103
	R: CTACCACTATCTTCGCCCTT		
*SED1*	F: CCACTGCTATCCCAACTAAC	50.5	110
	R: GGTTGGAGCTTCAGTAGTAG		
*ROM2*	F ATGGAGCAGGTCAAGTTATGG	54.7	99
	R: CAGCCCTGTTGGATGTATCTT		

**Table 2:** NCBI tBLASTn of *B. bruxellensis* LAMAP2480 sequence against *S. cerevisiae* Knr4p/Smi1p, Rom2p and Sed1p amino acid sequences.

Gen	*KNR4/SMI1*	*ROM2*	*SED1*
Description	Contig00356	Contig00135	Contig01043
Max score	177	786	39.3
Total score	177	786	78.9
Query cover	67%	69%	20%
*E value*	1 × 10^−47^	00.0	0.001
Percent identity	30.62%	45.08%	82.61%
Accession length	46050	15345	10582

## Data Availability

The datasets generated during and/or analyzed during the current study are available from the corresponding author on reasonable request.
